# Photobiomodulation Therapy in the Management of "Black Triangles" Due to the Absence of the Gingival Interdental Papilla

**DOI:** 10.7759/cureus.54682

**Published:** 2024-02-22

**Authors:** Marwan El Mobadder, Samir Nammour

**Affiliations:** 1 Department of Dental Sciences, University of Liege, Faculty of Medicine, Liege, BEL

**Keywords:** low-level laser therapy, periodontal regeneration, diode laser therapy, laser treatment, photobiomodulation, photobiomodulation therapy, papilla regeneration, gingival recession

## Abstract

The absence of an interdental papilla, termed "black triangle," presents a challenge in aesthetic restorations. Photobiomodulation therapy (PBMT) is the non-thermal therapeutic use of light in order to positively modulate biological activity and has shown promise in tissue regeneration, wound healing, and inflammation reduction. This case report introduces a modified PBM protocol known as "hemolasertherapy" aimed at regenerating the gingival interdental papilla to fill the black triangle. In this case report, a 34-year-old female with an unaesthetic black triangle between the maxillary central incisors presented for treatment. Before surgical intervention, our suggested protocol was proposed and explained, detailing potential risks and outcomes. After proper scaling root planning, a suggested protocol with PBM was made. PBM application with a 635 nm wavelength diode laser at four points around the area between the two maxillary central incisors was made: coronal third and apical third of the papilla and mesial and distal of the papilla. Then, bleeding was provoked with a curette inside the sulcus between 11 and 21 (concerned area) by applying pressure on the junctional epithelium and the supracrestal connective tissue. After a few seconds, blood spontaneously filled the “black triangle” coronal to the interdental papilla and was left undisturbed. At this point, PBM was applied again on the same four points already described. The irradiation parameters during all PBM treatments were contact mode and continuous mode, 635 nm wavelength, spot size of 8mm*, *power of 50 mW, irradiation time on each point of 50 seconds, and energy density of 4.976 J/cm^2^. After the intervention, the patient was prohibited from smoking, using mouthwash, drinking, and brushing for two hours. The exact same procedure was repeated five and 10 days after the first intervention. Follow-up was made for three months after the intervention. The assessment indicated a minor increase in the papilla height, which was not enough for a complete closure of the "black triangle." However, there was a reduction in the appearance of the black triangle. This case report suggests that PBM if used within our suggested protocol can increase the height of the gingival interdental papilla leading to a more pleasant aesthetic appearance. It is important to note that its effectiveness might be limited to specific conditions. In summary, the presented case report showcased a slight extension of the gingival interdental papilla. Further studies are essential to validate these observations.

## Introduction

In contemporary restorative dentistry, the primary aim is to attain aesthetic harmony in what are considered crucial zones, referred to as "white" and "pink" aesthetics. "White aesthetics" refers to the natural dentition or the repair of dental hard tissues using appropriate materials. On the other hand, "pink aesthetics" refers to the neighboring soft tissues, such as the interdental papilla and gingiva, which play a pivotal role in either elevating or detracting from the overall aesthetic outcome [1,2]. In this context, the presence or absence of an interdental papilla in the aesthetic zone either between implants or teeth gained attention in past years. In fact, the partial or total absence of an interdental papilla is now considered somehow a failure of the treatment in terms of aesthetics from both the patient and operator’s point of view. This has led to an increased demand for more approaches aimed at improving the aspect of interdental papilla, especially in the aesthetic zone [1].

The partial or complete absence of the gingival interdental papilla can be due to multiple factors [2,3]. These etiologies include dimensional changes in the interdental papilla during teeth alignment, gingival recession, periodontitis, destruction of the alveolar bone, the shape of the crowns, and the positioning of the inter-proximal contact point [2,3]. A classic study by Tarnow et al. [1] concluded that when the measurement between the contact point to the crestal bone is 5 mm or less, the papilla fills the space interdentally around 100% of the time. However, if the distance is above 6 mm, the interdental space is filled in around 55% of the time. At 7 mm and above, the concerned space is totally filled in only 25% of the time.

As for the management of the absent and/or partial absence of the interdental papilla, different approaches were described in the literature [3-5]. One such widely described option is the surgical reconstruction of the missing interdental papilla [6]. For instance, a sub-epithelial connective tissue graft with a coronally advanced flap proved to be a relatively successful approach for different types of papilla loss [7,8]. Nonetheless, surgical interventions require strict conditions, such as the presence of sufficient blood supply to the grafted tissue, to avoid small and confined scaffolds that might often hinder the success rates of the surgical procedures. In addition, these procedures require surgeons with skills and expertise in periodontal plastic surgery [7,8].

Photobiomodulation therapy (PBMT) previously known as low-level laser therapy is the non-thermal therapeutic utilization of light within red and near-infrared wavelengths to modulate biological activity [9-11]. In 2017, the North American Association of Laser Therapy (NAALT) and World Association of Laser Therapy (WALT) collectively established the nomenclature of PBM as the application of light in non-thermal mode for therapeutic purposes [12,13]. Recent advancements in technical, clinical, and photo-biological comprehension have propelled the rapid evolution of PBM [9-14]. For instance, numerous studies currently demonstrate the significant impact of PBM in reducing inflammation, alleviating pain, preventing fibrosis, stimulating the healing of the wound, and regenerating the tissues [11]. Despite the abundance of evidence supporting the therapeutic modifications induced by PBM in biological functions, the exact biological mechanisms underlying its effects remain incompletely understood [11]. These mechanisms vary depending on tissue conditions, cell types, irradiation parameters, and other influencing factors [11].

It is now well established that PBM enhances adenosine triphosphate (ATP) production and provokes a brief modulation of reactive oxygen species (ROS) [14,15]. The prevailing theory suggests that specific parameters of irradiation of light and/or infrared light stimulate cytochrome c oxidase (CcO), leading to increased ATP production [11]. Furthermore, current investigations propose that PBM may activate transcription factors and signaling pathways, suggesting potential protective mechanisms [11]. Hence, since PBMT has been proven to enhance ATP production, vasodilation, and notably tissue regeneration, it was suggested in this case report as a non-surgical method that can regenerate the gingival interdental papilla to fill the "black triangle." The protocol followed in this case report is a modification of a protocol suggested by Brugnera et al. under the nomenclature of "hemolaserherapy."

## Case presentation

Presentation and informed consent

**Figure 1:**
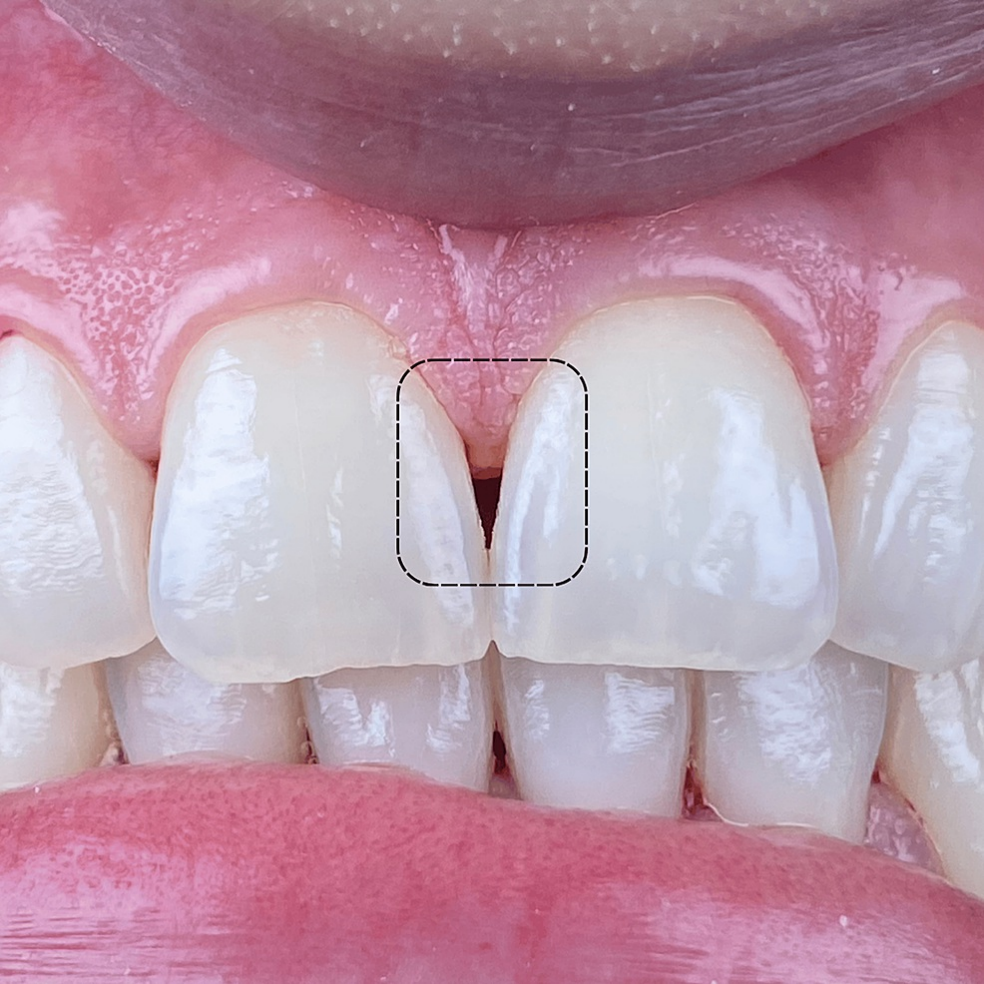
Clinical examination of the patient's maxillary arch emphasizes the presence of a black triangle coronal to the interdental papilla between the two maxillary central incisors.

**Figure 2:**
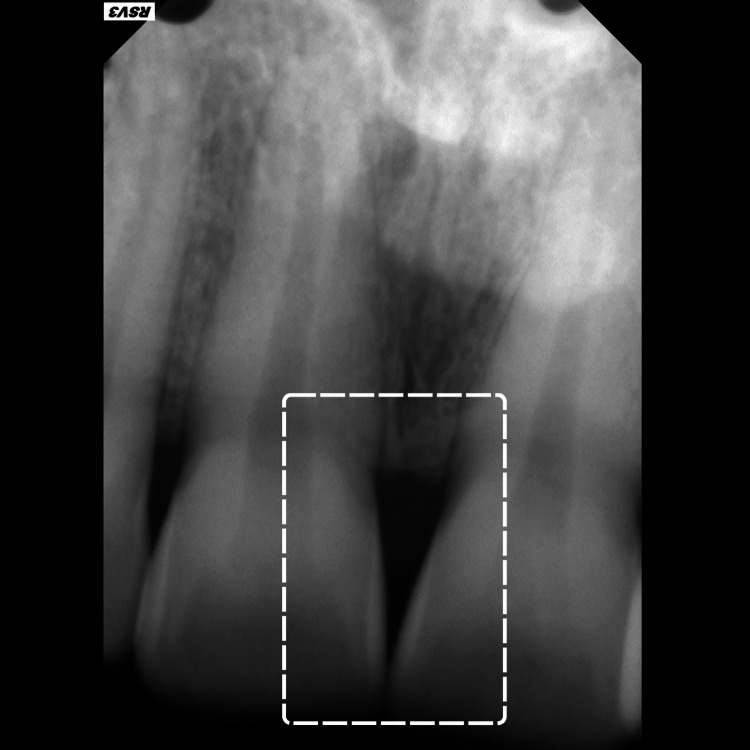
Peri-apical radiographs of the two central incisors illustrating the distance between the marginal alveolar bone and the contact point between the incisors.

A 34-year-old female patient presented to the clinic complaining of an unaesthetic aspect during her smile due to the black triangle coronal to the interdental papilla between the two maxillary central incisors (#11 and #21) (Figure 1). The patient is a light smoker (smokes shisha less than two times a week), with no systemic disease that can affect her periodontal or dental health. Before suggesting any surgical periodontal plastic surgery, our proposed protocol including PBM was suggested and explained to the patient for the management of her “black triangle” in the concerned area. The risks, potential side effects, and the possibility of no improvement after treatment were clearly explained. Afterward, the patient agreed to the treatment and signed a written informed consent before her enrollment. At this point, preoperative intra-oral photography (Figure 1) and peri-apical radiography were made (Figure 2).

The patient received oral hygiene instructions, including guidance on an adequate tooth brushing technique and the use of interdental brushes for interdental cleaning. Then, professional sub-gingival plaque removal was performed using an ultrasonic piezoelectric scaler for the entire mouth scaling root planning (SRP). Furthermore, instrumentation with curettes (universal and Gracey curettes) was made and chlorhexidine 0.12% (GUM PAROEX, 0,12% Intensive Action Mouthwash) solution was used to irrigate the sulcus for an average duration of almost 10 seconds using an endodontic syringe needle tip. The non-surgical treatment was made based on the recommendations of the European Federation of Periodontology (EFP). Afterward, with a periodontal probe (Hu-Friedy), the gap from the contact point to the peak of the alveolar bone was assessed with the application of local anesthesia, using pressure to reach the crestal bone. The noted distance was 6 mm. After one week of scaling root planning, there was no sign of any gingival inflammation and the periodontium was considered as healthy.

Treatment protocol

**Figure 3:**
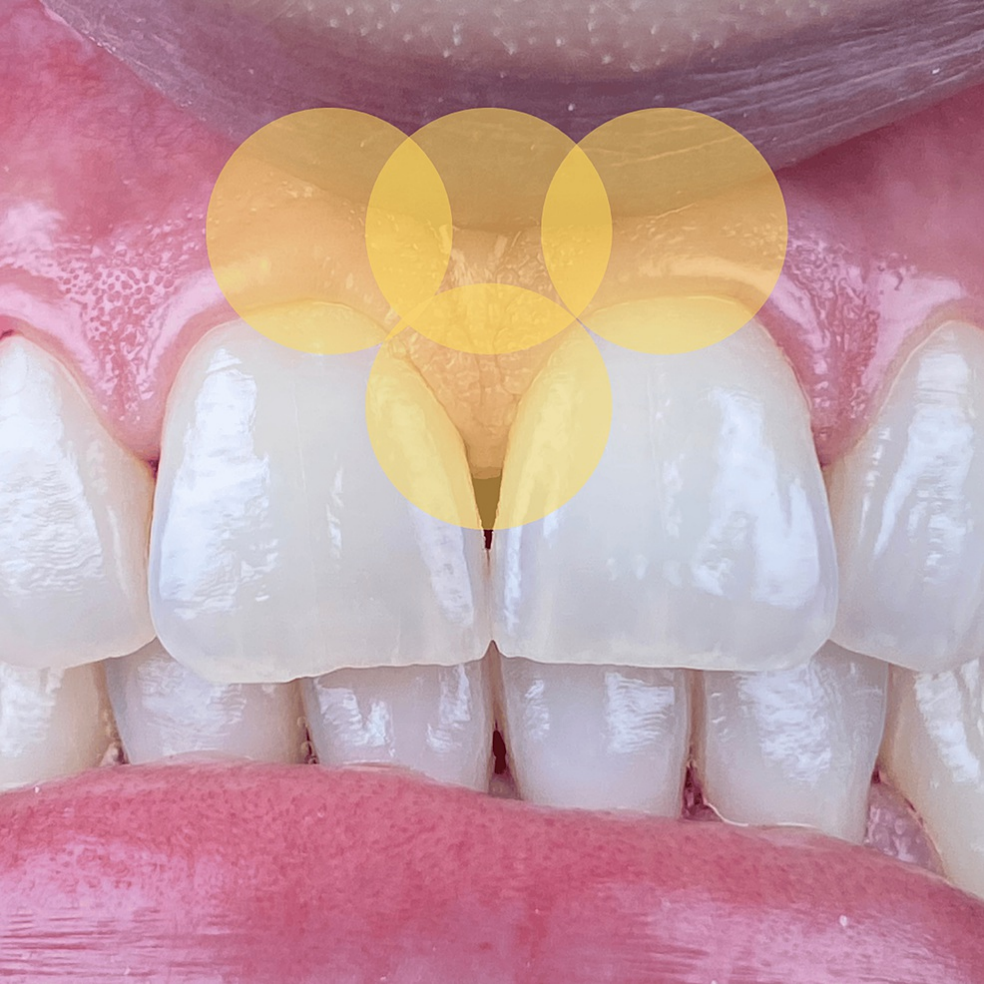
Illustration of the four points of photobiomodulation therapy applied on the concerned papilla.

**Figure 4:**
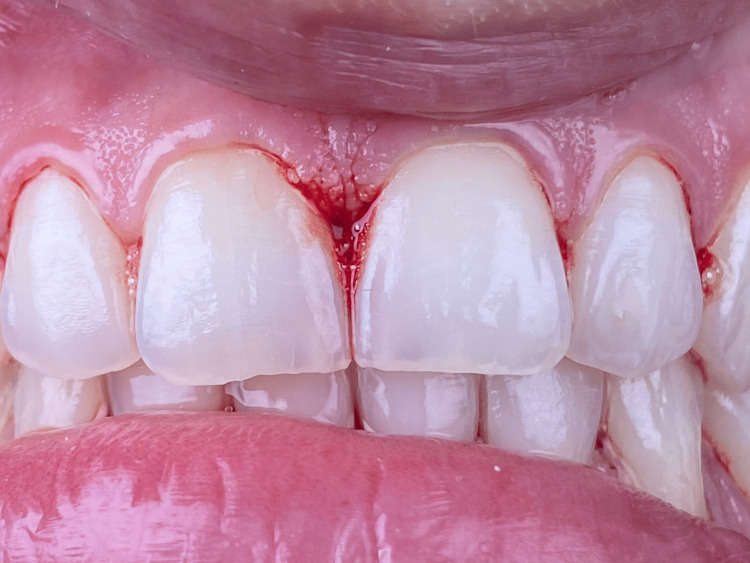
Aspect of the concerned area between the two maxillary central incisors after provoked bleeding and application of the PBM.

PBM within a specific protocol known as hemolasertherapy was followed with modifications. At the first session (T1), initial PBM application was made with a 635 nm wavelength diode laser (Smart M, Lasotronix, Warsaw, Poland) at four points around the area between the two maxillary central incisors: coronal third of the papilla, apical third of the papilla, and mesial and distal of the papilla (Figure 3). Later, bleeding was provoked inside the concerned sulcus (between 11 and 21) by applying pressure on the junctional epithelium and the supracrestal connective tissue using a curette (1/3 Gracey curettes, a premium instrument from the United Kingdom). After a few seconds, the stimulated blood spontaneously filled the “black triangle” coronal to the interdental papilla and was left undisturbed. At this point, PBMT was applied again on the same four points already described (Figure 4). Irradiation parameters during all PBM treatments were as follows: contact mode and continuous mode, 635 nm wavelength, spot size of 8 mm, power of 50 mW, irradiation time on each point of 50 seconds, energy of 2.5 J, and energy density of 4.976 J/cm^2^ (Table 1). After the intervention, the patient was prohibited from smoking for 48 hours, using any kind of mouthwash, and brushing for two hours. The exact same procedure was repeated five and 10 days after T1. The aspect of the newly formed gingival papilla and the presence or absence of the black triangle were evaluated at different times of follow-up. In addition, the height of this newly formed papilla was measured. Follow-up was made one month and three months postoperative.

**Table 1:** Photobiomodulation therapy irradiation parameters per point.

Parameters	Values
Wavelength	635 nm
Mode of delivery	Continuous mode
Irradiation mode	Contact mode
Power (per point)	50 mW
Irradiation time (per point)	50 seconds per point
Energy (per point)	2.5 J
Tip diameter (per point)	8 mm
Energy density (per point)	4.976 J/cm^2^

**Figure 5:**
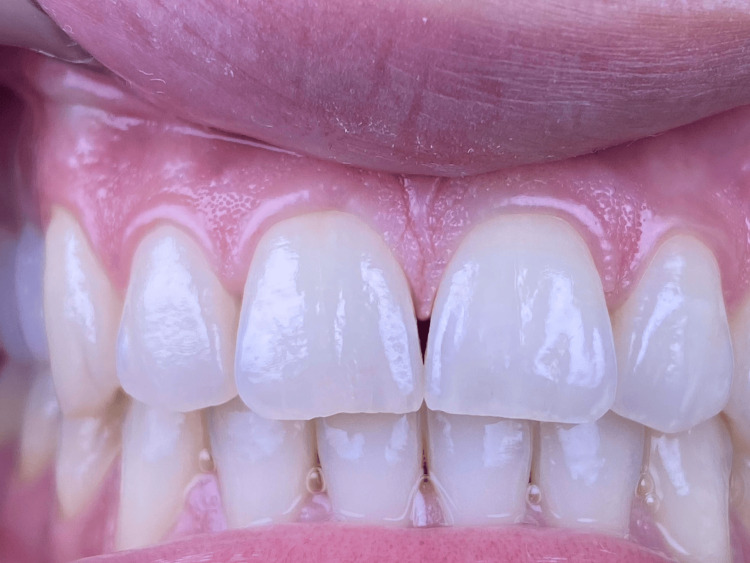
Aspect of the area between the two maxillary central incisors three months after the intervention.

Three months after the intervention, a clinical inspection of the interdental papilla between teeth #11 and #21 revealed a slight lengthening of the interdental papilla leading to a slight reduction of the black triangle. This suggests a possible reparation or "regeneration" of the gingival papilla (Figure 5). It is crucial to highlight that while the "black triangle" was not entirely absent, there was a slight improvement from an aesthetic point of view due an increase in the height of the interdental papilla.

## Discussion

In this specific case report, PBM was applied to target the "black triangle" between the two maxillary central incisors by promoting gingival papilla growth. After three sessions of the suggested protocol, an improvement in the papilla height was noted; however, it was not substantial enough to assert a complete closure of the black triangle or achieve entirely satisfactory results. Unlike the case report by Zanin et al. [11], in this study, we measured and reported the distance between the contact point of the two central incisors and the crest of the alveolar bone (6 mm). This enhances the predictability of further research within our protocol. In addition, while Zanin et al. [11] conducted two sessions, our protocol required three sessions to achieve what we considered necessary to achieve a significant stimulation of the interdental papilla.

Today, there is a plethora of clinical and laboratory research that supports the conclusion that PBM actively modulates biological functions [16-18]. Present data suggest that PBM acts predominantly on cytochrome c oxidase (CcO) in the mitochondrial respiratory chain by facilitating electron transport resulting in an increased transmembrane proton gradient that drives ATP production [18,19]. ATP is the universal energy source in living cells essential for all biological reactions, and even a small increase in ATP levels can enhance bioavailability to power the functions of cellular metabolism [9]. In addition, the adequate absorption of light within PBM resulted in a transient and short burst of reactive oxygen species (ROS), which is followed by an adaptive reduction in oxidative stress. This action, impairment of ROS production, has been suggested to mimic the activity of molecular agents that attenuate tissue damage (examples include amifostine, N-acetyl cysteine, and superoxide dismutase). Moreover, mild levels of ROS have significant implications on various cellular functions, including the activation of crucial transcription factors, like nuclear factor kappa B (NF-κB). Consequently, this activation leads to the expression of genes that stimulate and safeguard cellular growth, encompassing growth factors within the fibroblast growth factor family, pro-inflammatory cytokines, and chemokines essential for tissue repair [9]. Clinically, these demonstrated biological effects resulting in the facilitation of wound healing and tissue regeneration by influencing various stages of injury resolution. Moreover, PBM stimulates the proliferative phase after injury, which involves the activation of fibroblasts, macrophages, and other reparative elements, and the stimulation of the remodeling phase, characterized by collagen deposition and the reconstruction of the extracellular matrix in the vicinity of the wound [16-19].

Despite the mentioned well-documented activity of PBM, the slight improvement observed or lack thereof of a complete lengthening of the gingival papilla, as in this case, could be attributed to multiple factors that cannot be modified by PBMT. Such factors are anatomical factors, periodontal phenotype, the distance between the bone crest and contact point of the two central incisors [1], tooth form/shape, the curvature of the marginal gingiva, and the interproximal thickness of the gingiva [20]. For instance, the patient in this case report presented a distance between the contact point and the crestal bone that is more than 5 mm. This can be considered unfavorable based on the clinical study of Tarnow et al. [1]. For instance, for such cases, reconstructive periodontal plastic surgeries are usually indicated. Indeed, Tarnow et al. discovered that when the distance between the bone crest and the contact point was ≤5 mm, the papilla was present in 98% of cases. However, this occurrence decreased to 56% and 27% when the distance between the bone crest and the contact point reached 6 and 7 mm, respectively. Specifically, the proximity of the bone crest to the contact points (≤5 mm) and the thickness of the gingiva in the interproximal area (≥1.5 mm) strongly affect the presence of an interdental papilla, which are considered requirements for an ideal case [1]. Hence, this suggested treatment protocol yields a slight improvement in the papilla height and points out that PBMT can most likely stimulate the growth or “regeneration” of the interdental papilla to fill out the “black triangle,” which can be considered promising especially due to the unaggressive nature of PBM and the absence of any reported side effects. The protocol proposed herein can be regarded as a primary treatment option for papilla regeneration, addressing interdental papilla deficiency due to its non-invasive, non-surgical nature.

However, this study is on a single patient (case report); therefore, further research, especially randomized clinical trials, is imperative to validate the efficacy of such protocols. Moreover, the impact of this suggested protocol on different distances between the crest of the alveolar bone and the contact point should be investigated, as suggested by Tarnow et al. [1].

## Conclusions

This case report outlines a promising protocol comprising three sessions of PBMT followed by blood clot stimulation and subsequent photobiomodulation therapy. The suggested protocol demonstrated a modest increase in gingival interdental papilla length, resulting in a reduction in the overall size of the "black triangle" and thereby improving aesthetic outcomes. It is imperative to acknowledge the necessity for further investigations utilizing the proposed protocol on a larger cohort of patients, particularly in scenarios where the distance between the crestal bone and the contact point differs from that presented in this case report. Hence, within the limitations of this study, our suggested protocol can be taken into consideration as a non-invasive approach for managing the absence of the gingival interdental papilla. Therefore, within the limitations of this study, the proposed protocol can be regarded as a non-invasive approach for addressing the absence of the gingival interdental papilla.
